# Tumor Microenvironmental Regulation of CAR T-Cell Therapy in High Risk Medulloblastoma

**DOI:** 10.21203/rs.3.rs-9610745/v1

**Published:** 2026-05-21

**Authors:** Serge Yaacoub, Mostafa Seblani, Kaleem Coleman, Zhongzhen Yi, Kyoungtea Kim, Shang-Yang Chen, Bicna Song, Chao Di, Leon F. McSwain, Gage Paul, Olga Rodriguez, Chris Albanese, Alexander V. Kabanov, Christopher Lazarski, Timothy Gershon, Jo Lynne Rokita, Martine F. Roussel, Marina Sokolsky-Papkov, Dalia Haydar

**Affiliations:** 1 Center for Cancer and Immunology Research, Children’s National Hospital, Washington DC, USA 20010; 2 Center for Nanotechnology in Drug Delivery, Eshelman School of Pharmacy, University of North Carolina at Chapel Hill, Chapel Hill, North Carolina, 27599; 3 Department of Pediatrics, Emory University School of Medicine, Atlanta, GA, 30323; 4 Center for Translational Imaging, Georgetown Medical Center, Washington DC, 20007; 5 Department of Pediatrics, George Washington University School of Medicine, Washington DC 20052; 6 Department of Tumor Cell Biology, St Jude Children’s Research Hospital, Memphis, TN 38105

**Keywords:** Medulloblastoma, CAR T-cell therapy, tumor microenvironment, TLR7/8 agonism, myeloid immunomodulation, pediatric brain tumor immunotherapy

## Abstract

**Background:**

B7-H3-directed chimeric antigen receptor (CAR) T cell therapy has demonstrated clinical safety and antitumor activity in pediatric brain tumors (pBTs) but lacks durable responses. Although preclinical studies show efficacy, the CAR designs that best support sustained function in immunosuppressive tumor microenvironments (TMEs) remain unclear. Group 3 medulloblastoma (G3MB) is a lethal pBT with poor responsiveness to immunotherapy. Within the TME, myeloid cells dominate the immune landscape and regulate T cell function through innate immune pathways, including the Toll-like receptor 7/8 (TLR7/8) axis. While TLR7/8 agonists activate antitumor myeloid programs, their integration with CAR T-cell therapy has not been explored. Therefore, we hypothesize that CAR architecture and TLR7/8-mediated myeloid activation cooperatively govern CAR T-cell function and subsequent therapeutic outcomes in G3MB.

**Methods:**

B7-H3 CARs incorporating CD28, 4–1BB, or dual CD28/4–1BB co-stimulation were evaluated *in vitro* and in orthotopic G3MB models. Resiquimod was formulated in a brain-penetrant poly (2-oxazoline) nanoparticle (ResiPOx) to activate TLR7/8-expressing myeloid cells. T cell and myeloid states were assessed by flow cytometry, bulk, and single-cell RNA sequencing.

**Results:**

CAR designs showed similar tumor control in immunodeficient hosts but diverged in immunocompetent models, where dual-costimulatory CAR T-cells demonstrated superior cytotoxicity and persistence. The optimal CAR induced sustained CAR T-cell cycling and TAM reprogramming while downregulating TLR7/8 in dominant myeloid clusters. ResiPOx enhanced CAR T-cell efficacy by activating myeloid cells and reducing suppressive populations.

**Conclusions:**

Optimized CAR design combined with TLR7/8-mediated myeloid reprogramming enhances T cell activity, supporting TME-guided immunotherapy for G3MB.

## Importance of the study

Group 3 medulloblastoma remains among the deadliest pediatric brain tumors, and current CAR T-cell therapies have shown limited durability due to a myeloid-dominant, immunosuppressive tumor microenvironment. This study advances the field by defining a translationally feasible strategy that integrates CAR engineering with targeted microenvironmental modulation. Using immunocompetent models that more closely reflect human immune tumor microenvironment constraints, we identify a dual-costimulatory CAR architecture that improves antitumor activity and survival but also reveals adaptive suppression of innate sensing pathways in myeloid cells. To overcome this barrier, we pair CAR T-cells with resiquimod delivered in a clinically adaptable poly(2-oxazoline) nanoparticle formulation to activate innate immunity within the central nervous system. Together, these findings establish a rational combination approach linking a manufacturable CAR product with an adjunct immunomodulatory agent, providing a path toward preclinical safety studies and early-phase clinical testing for children with high-risk disease.

## Introduction:

Medulloblastoma is the most common malignant pediatric brain tumor (pBT) and comprises four molecular subgroups^1^. Group 3 medulloblastoma (G3MB) is the most aggressive, characterized by MYC- and GFI1-driven oncogenesis, rapid growth, and frequent metastatic dissemination^2^. Children with G3MB have particularly poor outcomes, with 5-year survival rates below 40%, underscoring an urgent need for improved therapeuties^3–5^.

Immunotherapy using chimeric antigen receptor (CAR) T-cells is of high interest for pBTs due to its potential to deliver tumor-specific and less toxic antitumor immune responses^4,6–8^. Adoptive T-cell therapy has achieved remarkable success in hematologic malignancies, validating engineered immune recognition as a powerful therapy^9^. However, despite strong preclinical performance, CAR T-cells have shown limited efficacy in solid and central nervous system (CNS) malignancies^7,10–12^.

B7-H3 is a compelling CAR target due to its broad and consistent expression across pBTs, including MB^13^. B7-H3-directed pediatric CAR T-cell trials have demonstrated feasibility, acceptable safety, and initial antitumor activity^13^. Nevertheless, durable responses remain uncommon, even with optimized antigen targeting, indicating that factors beyond target selection constrain therapeutic success in the CNS^10,12,14^.

Experience across CNS malignancies highlighted a distinct set of limitations to CAR T-cell efficacy, including impaired persistence, restricted trafficking and intratumoral distribution, and the physical and physiological constraints imposed by the blood–brain barrier and the immunosuppressive tumor microenvironment (TME)^7,12^. In G3MB, the TME is heavily enriched in tumor-associated macrophages (TAMs) and resident myeloid populations that actively suppress T-cell activation, proliferation, and effector functions^15–18^.

CAR design, from extracellular antigen binding domains to linkers and intracellular activation and costimulatory signaling modules, profoundly influences cytotoxicity, persistence, and CAR T-cell fate^19–22^. However, accumulating evidence indicates that CAR engineering alone is frequently insufficient to overcome myeloid-mediated immune suppression within the CNS^23–27^. Our work and that of others suggest that global myeloid depletion does not reliably relieve suppression and may impair CAR T-cell function, highlighting the need for targeted myeloid reprogramming^16,21,25,28,29^.

Innate immune sensing pathways offer a mechanistically rational avenue for reshaping suppressive myeloid states^30^. Toll-like receptor 7 and 8 (TLR7/8) signaling regulates inflammatory myeloid activation and is functionally active in medulloblastoma-associated myeloid populations^31–34^. Pharmacologic TLR7/8 agonists, including resiquimod (R848), have been shown to repolarize macrophages toward pro-inflammatory, antitumor phenotypes and enhance immune-mediated tumor control^33–37^.

Here, we report, to our knowledge, the first integrated strategy combining systematic optimization of B7-H3 CAR signaling with targeted reprogramming of the myeloid-dominant TME in G3MB. Using immunocompetent models, we identify a dual-costimulatory CAR design that enhances cytotoxicity, persistence, and survival, but also induces adaptive suppression of innate immune sensing in myeloid cells. We then demonstrate that TLR7/8 activation with Resiquimod reverses suppressive myeloid states, enhances intratumoral CAR T-cell function, and further extends survival. Together, these results establish CAR design and myeloid reprogramming as joint requirements for durable CAR T-cell efficacy in pBTs.

## Materials and Methods:

Detailed descriptions of experimental procedures are provided in the Supplementary Materials and Methods, including cell lines; human CAR T-cell generation and functional characterization; murine T-cell flow cytometry–based analyses; magnetic resonance imaging (MRI); *in vivo* bioluminescence imaging (IVIS); bone marrow–derived macrophage studies; synthesis and nanoformulation of resiquimod; flow cytometry (FACS); and single-cell RNA sequencing (scRNA-seq) analysis workflows.

### G3MB Model

The syngeneic G3MB model used in this study (G3MB#1232) was developed by Martine Roussel at St. Jude Children’s Research Hospital. This tumor cell line is characterized by MYC and GFI1 overexpression with wild-type Trp53, closely recapitulating the molecular features of high-risk pBTs. Endogenous B7-H3 expression in G3MB#1232 cells is approximately 30%; therefore, cells were genetically engineered to express higher levels of murine B7-H3, as previously described^21,38,39^ (**Supplementary Fig. 1A**).

G3MB#1232 cells were cultured as neurospheres in Neurobasal medium (Invitrogen, 21103–049) supplemented with B27 (Invitrogen, 17504–044), N2 (Invitrogen, 17502–048), human basic fibroblast growth factor (PeproTech, AF-100–18B), and human epidermal growth factor (PeproTech, AF-100–15). Cells were passaged using Accutase (Invitrogen, A11105–01).

### Murine CAR T-Cell Generation

Second-generation murine B7-H3 CARs were generated using retroviral vectors encoding an m276-derived scFv linked to either CD28- or 4–1BB-based costimulatory domains, as previously described^21^. T-cells were isolated from splenocytes of 6–8-week-old C57BL/6J-Thy1.1 mice and activated with anti-CD3/CD28 antibodies in complete RPMI supplemented with 10% FBS, GlutaMAX, penicillin/streptomycin, and IL-2 (50 U/mL). After 48 hours, T-cells were transduced with CAR-encoding retroviral supernatant on RetroNectin-coated plates. Transduced cells were harvested 48 hours later and expanded in IL-2–containing medium through day 7 post-transduction.

### *In Vitro* Functional Characterization

CAR T-cell cytotoxicity against G3MB#1232 cells was assessed using real-time live-cell imaging (xCELLigence, eLive, and eTox; Agilent Technologies, 8711003 and 8711009) and flow cytometry–based apoptosis and viability assays using Annexin V (BD Biosciences, 550474), anti-mouse CD3e (BD Biosciences, 562600), and Live/Dead Near-IR viability dye (Invitrogen, L34993).

Additional co-culture assays evaluated CAR T-cell cytolytic activity, proliferation, memory phenotype, and cytokine production. Levels of IL-2 (R&D Systems, D2050), IFN-γ (R&D Systems, MIF00), and TNF-α (R&D Systems, MTA00B) were quantified by ELISA.

### *In Vivo* Efficacy Studies

All animal procedures were conducted under an approved Institutional Animal Care and Use Committee (IACUC) protocol (IACUC #00030512) at the Children’s National Research Institute. Animals were monitored routinely and humanely euthanized upon reaching pre-established endpoints, including ≥20% weight loss, neurological symptoms, or per veterinary recommendation. C57BL/6J (Jackson Laboratory) and NOD scid gamma (NSG) mice, 4–5 weeks of age and of both sexes, were used for syngeneic and xenograft studies, respectively.

For orthotopic intracranial implantation, mice were anesthetized and secured in a stereotactic frame. A 1-mm burr hole was created, and 3 L of tumor cell suspension (murine or human-derived G3MB) was injected into the cerebellum at coordinates 2.5 mm posterior to lambda and 2.0 mm ventral from the skull surface. The incision was closed with wound clips. Fourteen days post-implantation, mice received intratumoral CAR T-cell infusion (1–2 × 10^6^ effector T-cells, normalized to 40% CAR expression) in 3 L of media using the same stereotactic coordinates. Studies were performed using CAR T-cells derived from independent biological donors.

Therapeutic efficacy was evaluated by survival monitoring. Tumor burden was assessed by MRI in syngeneic models and by IVIS in NSG xenografts, providing complementary assessments of tumor progression, antitumor activity, and CAR T-cell performance across immune contexts.

### Myeloid Immunomodulation Combined with CAR T-Cells

The impact of myeloid reprogramming in combination with B7-H3 CAR T-cells was evaluated by testing multiple treatment sequences relative to CAR T-cell infusion. Resiquimod was formulated in amphiphilic poly(2-oxazoline) nanoparticles (ResiPOx) and administered intraperitoneally on specified days following tumor implantation. Dosing regimens included single or multiple injections on days 13, 15, 17, and 19, relative to CAR T-cell infusion on day 14. This study design enabled assessment of how pre- versus post-CAR T-cell myeloid modulation influences CAR T-cell function.

### Immune Profiling of the TME

To evaluate TME remodeling, tumor-bearing brains were harvested at predefined time points classified as early (24h), intermediate (day 6), and endpoint. Cerebellar tissue containing tumor and adjacent normal brain was minced and enzymatically digested with collagenase (1 mg/mL) and DNase I (50 U/mL) for 60 minutes at 37 °C. Digests were mechanically dissociated and sequentially filtered through 70-m and 40-m strainers to generate single-cell suspensions, followed by ACK-mediated erythrocyte lysis and PBS washes. Additional filtration and low-speed centrifugation were applied as needed to reduce cellular debris.

CD45^+^ immune cells were enriched by magnetic selection using mouse CD45 MicroBeads (Miltenyi Biotec, 130-097-153). The CD45^−^ fraction was retained for downstream scRNA-seq. Samples were fixed and preserved for sequencing using the Chromium GEM-X Flex Sample Preparation v2 Kit (10x Genomics, PN 1000781). Additional details regarding TME characterization by FACS and scRNA-seq analyses are provided in the *Supplementary Methods*.

### Statistical Analyses

All experiments included at least three independent biological replicates, unless otherwise stated. Two-tailed Student’s *t* tests were used for comparisons between two groups. For comparisons involving three or more groups with a single independent variable, statistical significance was assessed using one-way ANOVA with Tukey’s multiple-comparisons test. For experiments involving two independent variables, multiple *t* tests or two-way ANOVA with Sidak’s or Tukey’s corrections were applied, as appropriate.

Survival curves were generated using the Kaplan–Meier method and compared using the log-rank (Mantel–Cox) test. IVIS data were analyzed using *t* tests or ANOVA, depending on the number of groups and experimental factors. All statistical analyses were performed using GraphPad Prism (RRID: SCR_002798). Statistical significance is denoted as follows: ns, not significant; *(P < 0.05); **(P < 0.01); ***(P < 0.001); *****(P < 0.0001). For clarity, non-significant comparisons are omitted from multi-comparison figures.

## Results:

### CAR Architecture Influences Efficacy in G3MB

To evaluate anti–B7-H3 CAR performance in G3MB, we first tested a previously optimized CAR incorporating CD28 costimulation and mutated CD3ζ ITAMs (^CD28TM^CD28.mutζ), which has demonstrated robust activity in glioma models^21^. *In vitro*, this CAR mediated potent, antigen-dependent cytotoxicity against G3MB#1232 tumor spheroids, as measured by real-time xCELLigence live-cell imaging, whereas control CAR T-cells showed no detectable cytotoxic activity (**Supplementary Fig. 1B–C**).

Despite strong *in vitro* efficacy, intracranial administration of ^CD28TM^CD28.mutζ CAR T-cells 14 days after G3MB#1232 implantation in immunocompetent C57BL/6 mice did not result in durable antitumor control. MRI demonstrated transient tumor stabilization as early as day 6 following CAR T-cell treatment (**Supplementary Fig. 1D-E**); however, radiographic progression was evident by days 16 and 25 in most of the animals (**Supplementary Fig. 1E**). Consistent with these findings, overall survival was not significantly improved compared with control CAR treatment (**Supplementary Fig. 1F**), indicating limited long-term efficacy of this CAR design in G3MB.

### Optimizing CAR Architecture for G3MB

Given the lack of durable antitumor efficacy observed with the prototype CAR in G3MB, we next sought to optimize CAR signaling architecture. We evaluated a panel of murine B7-H3–directed CAR constructs incorporating different transmembrane and costimulatory domains^21^ (**Supplementary Fig. 2A**). This panel included the ^CD28TM^CD28.mutζ CAR, a signaling-deficient control CAR, CARs incorporating either CD28 or CD8 transmembrane domains paired with a 4–1BB costimulatory domain and mutated CD3ζ (^CD28TM^41BB.mutζ and ^CD8TM^41BB.mutζ), and a dual-costimulatory configuration achieved by expression of 4–1BB ligand *in trans* on ^CD28TM^CD28.mutζ CAR T-cells (41BBL-^CD28TM^CD28.mutζ).

CAR T-cells were generated by retroviral transduction (**Supplementary Fig. 2B**). Although CAR expression levels varied across constructs (**Supplementary Fig. 2C**), products were normalized to achieve 40–60% CAR positivity prior to functional evaluation (**Supplementary Fig. 2D**). Phenotypic analyses revealed a consistent enrichment of CD8^+^ T-cells across all CAR constructs (*P* < 0.0001; **Supplementary Fig. 2E-i**). Within the CD4^+^ compartment, central memory phenotypes predominated across most constructs (*P* < 0.0001), with relative reductions observed in ^CD28TM^CD28.mutζ and 41BBL-^CD28TM^CD28.mutζ CAR T-cells (*P* < 0.05 and ns, respectively; **Supplementary Fig. 2E-ii**). In the CD8^+^ compartment, effector versus central memory distributions were largely comparable across constructs, although ^CD28TM^CD28.mutζ and 41BBL-^CD28TM^CD28.mutζ CAR T-cells exhibited modest skewing toward effector memory phenotypes (*P* < 0.05; **Supplementary Fig. 2E-iii**).

Expression of exhaustion markers, including PD-1, LAG-3, and TIM-3, remained low across constructs in both CD4^+^ and CD8^+^ CAR-positive populations (**Supplementary Fig. 2F**). A modest but statistically significant increase in PD-1 expression was observed in ^CD28TM^CD28.mutζ CAR T-cells compared with control CAR T-cells (*P* < 0.05).

### Dual-Costimulatory CARs Exhibit Enhanced Cytotoxic Kinetics *In Vitro*

**Figure 1.**
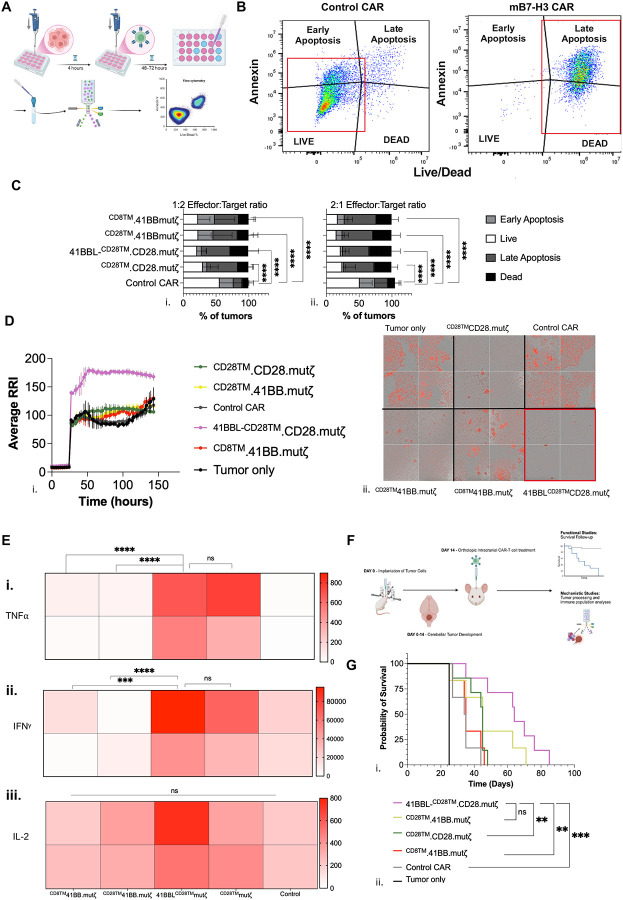
Functional characterization of murine B7-H3 CAR T-cells with distinct CAR designs *in vitro* and *in vivo*. A. Schematic of the *in vitro* flow cytometry–based coculture assay and apoptotic profiling workflow; created with Biorender.com. Tumor cells were plated and allowed to establish for 4 hours before addition of CAR T-cells, followed by 48–72 hours of coculture prior to flow cytometric analysis. *Created in BioRender. Yaacoub, S. (2026)* https://BioRender.com/zasdf76 B. Representative Annexin V versus live/dead plots for Control CAR and mB7-H3 CAR conditions are shown C. Tumor cells were classified as live, early apoptotic, late apoptotic, or dead based on Annexin V and live/dead staining. Stacked bar plots summarize the distribution of tumor cell states across CAR constructs under the indicated coculture conditions (i- 1:2 ii-2:1 ratios) *(n = 13, mean SD, two-way ANOVA with Tukey test for multiple comparisons)*. D. Real-time cytotoxicity analysis of murine CAR T-cells against G3MB tumor cells using the xCELLigence platform. i. Tumor killing kinetics are shown as average relative red intensity of the ETOX Red dead-cell signal over time for the indicated CAR designs, Control CAR, and tumor-only conditions, reflecting differential cytotoxic activity across constructs. ii. Representative images of Etox signal from xCELLigence assay evaluating different CAR constructs against G3 MB cells. Etox signal highlights dying cells, with dark red staining indicating dead tumor cells at the 92 hr mark. 4–1BBL-harboring B7-H3 CAR T-cells exhibited the fastest cytotoxic effects and most longstanding residual tumor clearance across different B7-H3 CARs. E. Heatmaps showing cytokine release across murine CAR designs, including IL-2 (i), IFN-γ (ii), and TNF-α (iii), measured by ELISA following CAR T cell–tumor coculture. Each column represents an individual CAR construct or control condition, and color intensity indicates relative cytokine abundance. For each heatmap, the top row represents the 2:1 CAR T cell–to–tumor cell ratio, and the bottom row represents the 1:2 ratio. (n=8 per ELISA data set condition; two-way ANOVA with Sidak’s test for multiple comparisons; * P < 0.05; ** P < 0.01; *** P < 0.001; **** P < 0.0001; ns, non-significant.). F. Schematic of the orthotopic intracranial medulloblastoma model used for *in vivo* CAR T-cell studies, including tumor implantation at day 0, CAR T-cell treatment at day 14, and downstream survival and mechanistic analyses G. Kaplan–Meier survival curves comparing tumor-only, Control CAR, and mB7-H3 CAR treatment groups are shown. (n=6 per treatment group; survival curves were determined using the log-rank (Mantel-Cox) test. * p<0.05; ** p<0.01; *** p<0.001; **** p<0.0001; ns, non-significant.)

To compare cytotoxic activity across CAR architectures, CAR T-cells were co-cultured with G3MB spheroids for 72 hours at effector-to-target (E:T) ratios of 2:1 and 1:2. Tumor cell apoptosis and death were quantified by FACS following gating on CD3^−^ cells (Fig. 1A–B). All functional B7-H3 CARs mediated significant antigen-dependent tumor killing compared with the control CAR (*P* < 0.0001), even at low E:T ratios, confirming potent *in vitro* cytotoxicity (Fig. 1C). Notably, endpoint levels of cytotoxicity were comparable across CAR designs.

To resolve potential architectural differences in killing dynamics, we next assessed cytotoxic kinetics using real-time xCELLigence assays incorporating Agilent eTox Red dye to track tumor cell clearance over time (Fig. 1D). These analyses revealed that the dual-costimulatory 41BBL-^CD28TM^CD28.mutζ CAR mediated the most rapid and robust tumor cell elimination, whereas other functional CARs exhibited slower kinetics but ultimately achieved comparable levels of tumor clearance (Fig. 1D–i). Representative xCELLigence images demonstrated reduced spheroid persistence and accelerated tumor elimination with the 41BBL-^CD28TM^CD28.mutζ CAR (Fig. 1D–ii).

Consistent with enhanced cytotoxic kinetics, ELISA analysis of co-culture supernatants collected at 24 hours revealed increased secretion of pro-inflammatory cytokines by 41BBL-^CD28TM^CD28.mutζ CAR T-cells, particularly IFN-γ and TNF-α (*P* < 0.001 and *P* < 0.0001, respectively), with more modest effects observed for IL-2 (Fig. 1E).

### 41BBL-^CD28TM^CD28.mutζ CAR T-Cells Exhibit Superior Efficacy in Syngeneic G3MB

We next evaluated the impact of CAR signaling architecture on antitumor efficacy within an immunocompetent TME using the syngeneic G3MB#1232 model (Fig. 1F). Comparison of B7-H3–directed CAR designs revealed marked construct-dependent differences in therapeutic performance. Notably, the dual-costimulatory 41BBL-^CD28TM^CD28.mutζ CAR mediated superior tumor control and significantly prolonged survival relative to all other CAR configurations. Median survival in the 41BBL-^CD28TM^CD28.mutζ group was 64 days, compared with 45 days for ^CD28TM^CD28.mutζ and ^CD28TM^41BB.mutζ, 35 days for ^CD8TM^CD28.mutζ, 34 days for the control CAR, and 25 days for untreated mice. Survival was significantly improved in the 41BBL-^CD28TM^CD28.mutζ group compared with most other treatment groups (log-rank test, *P* < 0.01) (Fig. 1G).

Collectively, these findings demonstrate that CAR signaling architecture is a critical determinant of therapeutic efficacy in immunocompetent G3MB, with dual costimulation conferring a significant survival advantage consistent with enhanced cytotoxic kinetics, persistence, and *in vivo* function.

### CAR Design–Dependent Efficacy Is Lost in the Absence of an Intact TME

To determine whether CAR architecture–dependent differences in efficacy were contingent on interactions with the TME, we next evaluated human B7-H3 CAR T-cells in immunodeficient NSG mice lacking an intact immune TME. Human CAR T-cells were generated from activated peripheral blood mononuclear cells using a panel of human-derived CARs analogous to those tested in murine models (**Supplementary Fig. 3A–B**). CAR transduction efficiency and phenotypic profiles were confirmed by FACS (**Supplementary Fig. 3C–D**).

*In vitro* cytotoxicity assays against D556 G3MB demonstrated modestly enhanced tumor killing by the 41BBL-expressing CAR (*P* < 0.0001), although all functional CARs mediated significant cytotoxicity compared with control CAR T-cells (**Supplementary Fig. 3E**). These findings were reproduced in a second G3MB model (MB002) using FACS–based Annexin V apoptosis assays (**Supplementary Fig. 3F**). Consistent with these results, cytokine analyses of coculture supernatants revealed comparable IL-2 and IFN-γ secretion across CAR constructs (**Supplementary Fig. 3G**), whereas MB002 cocultures demonstrated significantly increased IL-2 secretion by the 41BBL-expressing CAR (*P* = 0.0003), with no differences in IFN-γ production (**Supplementary Fig. 3H**).

In contrast to findings in immunocompetent models, *in vivo* evaluation in NSG mice revealed no significant survival advantage among human CAR designs following intratumoral delivery of 2 × 10^6^ CAR T-cells (**Supplementary Fig. 4A**). Although modest differences in early tumor burden were detected by IVIS (**Supplementary Fig. 4B**), CAR signaling architecture did not translate into meaningful differences in survival outcomes. Collectively, these data indicate that CAR architecture–dependent differences in therapeutic efficacy are attenuated in immunodeficient settings, supporting a critical role for immune-competent TME interactions in mediating *in vivo* performance of different CAR designs.

### CAR architecture reshapes intratumoral immune networks and T-cell fate states in immunocompetent G3MB

**Figure 2.**
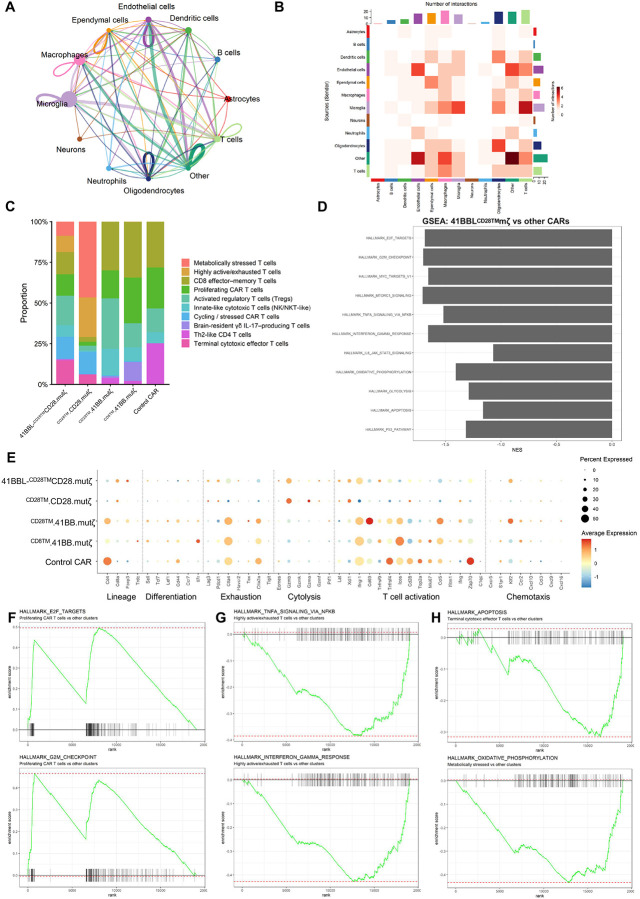
Transcriptional heterogeneity of tumor-infiltrating T-cell populations across treatment conditions. A. Circular network plot depicting inferred ligand–receptor interactions among malignant cells, CNS-resident populations, and infiltrating immune lineages within intracranial tumors. Edge thickness reflects the relative number of predicted interactions between cell types. B. Heatmap summarizing the strength and frequency of predicted intercellular interactions between immune populations across CAR treatment conditions, highlighting differential engagement of myeloid and lymphoid compartments. C. Stacked bar plots showing the proportional distribution of CAR T-cell transcriptional states across experimental groups, including proliferative, activated, stressed, metabolically dysregulated, and terminal cytotoxic effector states. D. Summary bar plot of Hallmark gene set enrichment analysis (GSEA) across CAR T-cell states, displaying normalized enrichment scores (NES) for representative biological pathways. E. Dot-plot visualization of state-defining marker genes across CAR T-cell clusters, with dot size indicating the percentage of cells expressing each gene and color representing average expression. F. Representative GSEA enrichment plots demonstrating positive enrichment of cell-cycle programs (E2F targets and G2M checkpoints)in proliferating CAR T-cell states. G. GSEA enrichment plots illustrating negative enrichment of inflammatory effector pathways, including TNF α signaling, NFK-β, and IFN-y response, in highly activated or dysfunctional CAR T-cell states relative to other clusters. H. Additional GSEA enrichment plots showing negative enrichment of survival- and metabolism-associated pathways, including apoptosis and oxidative phosphorylation in terminal cytotoxic effector CAR T-cell states, consistent with state-dependent attenuation of effector and metabolic programs at terminal endpoints.

To define how CAR signaling architecture remodels the TME, we scRNA-seq on tumors harvested at an intermediate post-treatment time point (day 6), a window in which CAR T-cells are expected to exert major biological effects. We applied CellChat to infer ligand–receptor communication^40^. This analysis revealed dense intercellular connectivity across immune, stromal, and neural compartments, with prominent interaction hubs involving myeloid populations and extensive crosstalk between T-cells and microglia/macrophages/monocytes (Fig. 2A–B). These findings support a model in which CAR efficacy in immunocompetent G3MB is coupled to broader, network-level immune remodeling rather than exclusively CAR T-cell–intrinsic effects.

Unsupervised subclustering of tumor-infiltrating T-cells identified multiple transcriptionally distinct lymphoid states spanning proliferative, effector-memory, terminal cytotoxic, regulatory, and stress/dysfunction programs (**Supplementary Fig. 5A**). Dot-plot marker analyses confirmed lineage-defining transcriptional programs across these states and supported the integrity of cluster annotation (**Supplementary Fig. 5B**).

While most states were detectable across conditions, their relative abundance differed by CAR construct (Fig. 2C), consistent with the construct-dependent divergence in therapeutic performance observed in the immunocompetent model. In particular, the top-performing 4–1BBL–expressing CAR was associated with increased representation of terminal cytotoxic effector T-cells. In contrast, the intermediate-performing ^CD28TM^CD28.mutζ CAR showed a higher proportion of metabolically stressed and highly activated/dysfunctional states. Other functional CARs incorporating 4–1BB costimulation, as well as control CARs, exhibited redistribution toward proliferative, regulatory, and stress-associated states, with comparatively limited accumulation of cytotoxic/effector compartments (Fig. 2C).

To define construct-associated transcriptional programs, we performed Hallmark gene set enrichment analysis (GSEA) comparing the 41BBL-^CD28TM^CD28.mutζ CAR condition to the remaining CAR designs. Multiple pathways showed negative normalized enrichment scores (NES) in the 41BBL-^CD28TM^CD28.mutζ group (Fig. 2D), indicating relative depletion of these programs compared with other CAR designs. Notably, superior efficacy correlated with selective immune rewiring rather than maximal inflammatory activation, including relative suppression of interferon/inflammatory Hallmark programs in the 41BBL-^CD28TM^CD28.mutζ condition (Fig. 2D).

We next summarized lineage, differentiation, exhaustion, cytolysis, activation, and chemotaxis marker modules across treatment groups. This analysis demonstrated qualitative, construct-dependent differences in functional programming (Fig. 2E), aligning with observed shifts in overall T-cell state composition. Across conditions lacking durable benefit, module patterns were more consistent with dysfunctional activation and stress-associated states, whereas the 41BBL-^CD28TM^CD28.mutζ CAR displayed a profile more compatible with productive activation and differentiation rather than sustained dysfunctional activation (Fig. 2E).

### Transcriptional programs define intratumoral CAR T-cell functional states

To refine the functional interpretation of intratumoral CAR T-cell states, cluster-resolved GSEA revealed distinct, directionally opposed transcriptional programs within the TME. Proliferative CAR T-cell states were defined by canonical cell-cycle activity, with positive enrichment (NES > 0) of E2F target and G2M checkpoint gene sets in CAR-expressing T-cells relative to other T-cell clusters (Fig. 2F), consistent with active proliferation within the CNS niche.

In contrast, highly activated or dysfunctional CAR T-cell states exhibited negative enrichment (NES < 0) of inflammatory effector programs, including TNFα/NFκB signaling and interferon-γ responses (Fig. 2G). This relative depletion of effector signaling is consistent with attenuation of productive inflammatory programs at a terminally constrained or dysfunctional endpoint rather than sustained activation.

Terminal cytotoxic effector CAR T-cell states were characterized by negative enrichment of apoptosis and oxidative phosphorylation pathways (Fig. 2H), indicating a relative loss of survival- and metabolism-associated programs compared with other T-cell states. Additional Hallmark analyses further supported state-specific transcriptional divergence across CAR T-cell populations. Cycling or stress-associated CAR T-cell states showed enrichment of DNA damage response, glycolytic, and reactive oxygen species pathways, consistent with metabolic and replicative stress rather than effective cytotoxic function (**Supplementary Fig. 6A, 7B**). By contrast, highly activated or exhausted states exhibited attenuation of interferon-γ and TNFα/NFκB signaling alongside enrichment of apoptotic signatures, suggesting progression toward dysfunction (**Supplementary Fig. 6B**). Terminal effector states similarly displayed negative enrichment of oxidative phosphorylation and survival pathways, consistent with terminal differentiation under intracranial constraints (**Supplementary Fig. 7A**).

Collectively, these data demonstrate that intratumoral CAR T-cell functional states are organized along opposing transcriptional axes, with proliferative programs occupying one pole and terminal or dysfunctional programs defined by attenuation of inflammatory, metabolic, and survival pathways occupying the other. These direction-dependent, construct-associated transcriptional features support a model in which CAR design influences therapeutic efficacy through state-specific network remodeling within the brain TME, rather than through sustained inflammatory activation alone.

### Distinct CAR designs drive divergent TME remodeling in immunocompetent G3MB

Given the construct-dependent differences observed among intratumoral CAR T-cell functional states, we next examined whether the myeloid-dominant TME undergoes parallel, CAR-specific remodeling. Integrated myeloid subclustering combined with compositional and pathway analyses identified distinct immune and structural brain cell populations and revealed statistically supported redistribution and transcriptional reprogramming of microglial and TAM states across CAR designs (**Supplementary Fig. 8**).

scRNAseq analysis of G3MB tumors resolved a complex neuro-oncologic ecosystem comprising malignant cells alongside resident and infiltrating stromal and immune populations, including astrocytes, oligodendrocytes, microglia, peripheral myeloid subsets, and lymphocytes (**Supplementary Fig. 8A**). Canonical marker expression enabled robust lineage annotation across clusters. Subsequent enrichment of CD45^+^ immune compartments and dataset harmonization revealed a diverse immune landscape on UMAP projection, with discrete representations of microglia, macrophages, monocytes, dendritic cells, neutrophils, and lymphocytes (**Supplementary Fig. 8A–B**). Quantitative analyses demonstrated CAR-dependent remodeling of the intracranial TME, characterized by shifts in glial and myeloid compartmentalization and changes in immune subset proportions across functional CARs compared with tumor-only and control CAR (**Supplementary Fig. 8C**). Consistent with these compositional changes, CellChat analyses revealed dynamically altered intercellular communication networks among immune and non-immune cell populations in a CAR-specific manner, indicating coordinated reprogramming of intratumoral signaling networks rather than uniform immune activation (**Supplementary Fig. 9**).

### CAR designs impart distinct and functionally divergent myeloid remodeling across the TME

**Figure 3.**
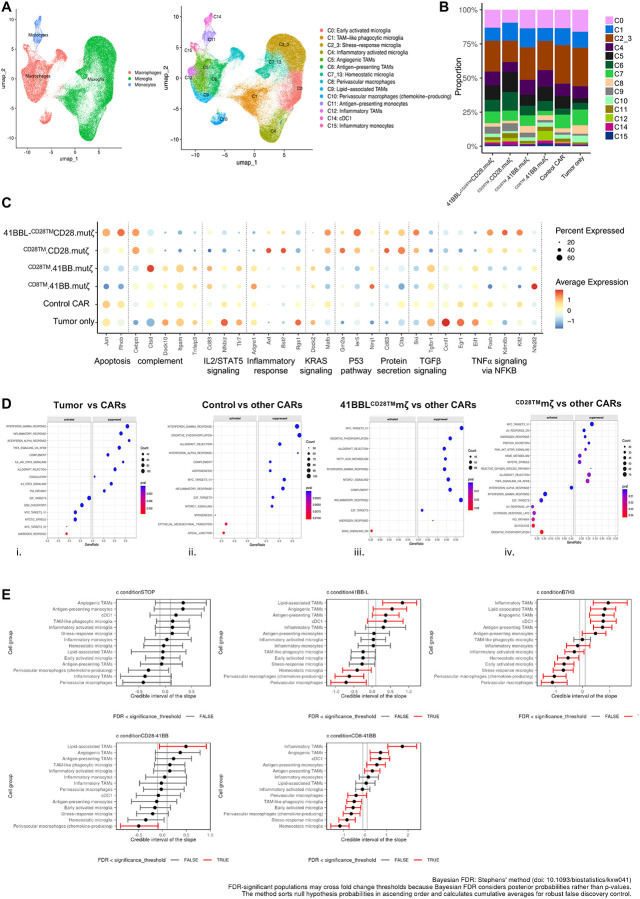
CAR-dependent remodeling of the intratumoral myeloid compartment in intracranial tumors A. UMAP projection of CD45^+^ myeloid cells isolated from intracranial tumors, showing separation of major myeloid populations, including microglia, macrophages, and monocytes. Moreover, exhibits of higher-resolution UMAP of myeloid cells colored by refined subcluster annotation, resolving resident microglia, infiltrating macrophage subsets, inflammatory monocytes, antigen-presenting populations, and proliferative myeloid states based on canonical marker expression. B. Stacked bar plots depicting the proportional distribution of annotated myeloid subclusters across experimental conditions, illustrating treatment-associated shifts in myeloid composition following CAR T-cell therapy relative to control and tumor-only groups. C. Dot-plot representation of state-defining genes across myeloid subclusters and treatment groups, with dot size indicating the percentage of cells expressing each gene and color representing average expression, highlighting functional programs associated with activation, antigen presentation, inflammation, and immunoregulation. D. Gene set enrichment analysis (GSEA) dot plots comparing tumor-only versus CAR-treated conditions, control CAR versus other CAR constructs, and individual CAR designs versus all other CARs, demonstrating construct-specific enrichment of myeloid functional pathways. E. Forest plots summarizing differential abundance analysis of myeloid populations across pairwise comparisons, with effect sizes and confidence intervals shown for each subpopulation and false discovery rate (FDR) thresholds indicated, identifying CAR-dependent expansion or contraction of specific myeloid states within the intracranial TME.

To determine how CAR T-cell therapy reshapes the myeloid compartment, we performed high-resolution subclustering of intratumoral microglia and macrophages. This analysis revealed multiple transcriptionally distinct myeloid states spanning early activated microglia, stress-responsive microglia, inflammatory microglia, TAM–like phagocytes, angiogenic TAMs, inflammatory monocytes, and related subsets (Fig. 3A–B). Importantly, projection of treatment groups onto this embedding demonstrated that CAR T-cell therapy does not uniformly activate myeloid cells, but instead redistributes them into distinct functional states in a CAR-design–dependent manner (**Supplementary Fig. 10A**).

In untreated tumors and control CAR conditions, the myeloid compartment remained dominated by immunosuppressive, M2-like and recruitment-associated states consistent with a baseline tumor-supportive microenvironment. By contrast, functional CARs disrupted this suppressive equilibrium, although the nature of myeloid remodeling differed substantially across constructs. Dot-plot analyses confirmed clear transcriptional distinctions between resident microglia and infiltrating macrophage and monocyte populations, enabling resolution of programs associated with immunosuppression, immune recruitment, invasion, and polarized M1-like versus M2-like states (**Supplementary Fig. 10B–C**). Collectively, these data indicate that CAR T-cell therapy reshapes the composition and polarization of tumor-associated myeloid cells rather than simply increasing global inflammation.

Comparative gene-expression analyses further clarified these differences. Untreated tumors were enriched for immunosuppressive, M2-associated programs with relative suppression of inflammatory and immune-recruitment pathways (Fig. 3C). CAR T-cell therapy broadly reduced suppressive myeloid signatures and induced genes linked to immune activation and recruitment; however, the quality and balance of this remodeling depended strongly on CAR architecture (Fig. 3C; **Supplementary Fig. 11A–B**). Notably, ^CD8TM^41BB.mutζ CARs induced M1-like transcriptional features yet retained select immunosuppressive programs, suggesting partial or functionally uncoupled activation. In contrast, control CARs elicited minimal inflammatory remodeling while preserving recruitment-associated responses characteristic of a non-productive immune milieu (Fig. 3C).

To define the transcriptional logic underlying these shifts, we performed GSEA across myeloid clusters. Control CAR treatment downregulated innate immune regulators, including *Il12rb1* and *Lbp*, and failed to induce inflammatory signaling pathways, consistent with minimal myeloid reprogramming (Fig. 3D; **Supplementary Fig. 11C**). ^CD28TM^CD28.mutζ CAR treatment instead drove an intermediate myeloid state characterized by enrichment of hypoxia- and interferon-α–associated programs together with suppression of MYC-driven and angiogenic pathways, reflecting stress- and proliferation-linked remodeling rather than coordinated inflammatory activation (Fig. 3D; **Supplementary Fig. 11C**).

Strikingly, the top-performing 41BBL-^CD28TM^CD28.mutζ CAR induced a selective and non-hyperinflammatory myeloid transcriptional program. This program was marked by upregulation of *S1pr4* and downregulation of prostaglandin signaling and cellular trafficking genes, consistent with targeted dismantling of suppressive signaling rather than broad inflammatory activation (Fig. 3D; **Supplementary Fig. 11C**). Pathway-level analyses supported this interpretation, revealing relative suppression of interferon- and inflammation-associated Hallmark programs in the 41BBL-^CD28TM^CD28.mutζ condition despite superior therapeutic outcomes. Compared with other CARs, the ^CD8TM^41BB.mζ condition shows a restrained myeloid landscape transcriptional shift marked by selective upregulation of macrophage-associated genes and modest activation of inflammatory and interferon-γ response pathways, alongside suppression of proliferative and metabolic programs (**Supplementary Fig. 12A)**. In contrast, ^CD28TM^41BB.mζ condition associated with a broader and stronger myeloid transcriptional reprogramming characterized by widespread downregulation of interferon, inflammatory, TNF–NFκB, and hypoxia signaling pathways, indicating a more globally suppressed inflammatory myeloid landscape (**Supplementary Fig. 12B)**.

Finally, forest-plot analyses demonstrated structured, CAR-dependent expansion and contraction of defined myeloid populations within the TME (Fig. 3E). Significant changes were observed across multiple antigen-presenting and inflammatory myeloid states, whereas other resident or peripheral subsets remained stable. Together, these findings indicate that effective CAR T-cell therapy reshapes the myeloid compartment through selective rewiring of suppressive and regulatory programs, rather than through indiscriminate activation, and that CAR signaling architecture is a key determinant of this process.

### CAR-dependent remodeling of dendritic cell (DC) states in the TME

To determine whether CAR T-cell therapy reshapes the antigen-presenting compartment of the TME, we performed focused single-cell analysis of intratumoral DCs. Unsupervised clustering resolved multiple transcriptionally distinct DC states, including conventional cDC1 and cDC2 populations, migratory DCs, and an inflammatory IL-12–associated DC subset, demonstrating marked functional heterogeneity within G3MB (**Supplementary Fig. 13A**).

CAR T-cell therapy altered the composition and distribution of these DC states in a CAR-design–dependent manner. Quantitative analyses showed that effective CAR constructs increased the relative abundance of inflammatory and migratory DC populations compared with tumor-only and control CAR conditions, which remained dominated by steady-state or non-inflammatory DC states (**Supplementary Fig. 13B**). Visualization of individual treatment groups on shared UMAP embeddings further demonstrated construct-specific restructuring of the DC landscape rather than uniform expansion across states (**Supplementary Fig. 13C**).

Functional profiling of DC subclusters using Hallmark pathway AUCell analysis revealed clear transcriptional polarization between DC states enriched for inflammatory, interferon, cytokine signaling, and antigen-presentation programs and those characterized by metabolic, stress-associated, or regulatory pathways (**Supplementary Fig. 13D**). CAR designs associated with superior therapeutic efficacy preferentially supported DC states aligned with immune activation and coordination, linking CAR signaling architecture to remodeling of the dendritic cell compartment within the CNS tumor microenvironment.

### TLR7/8-associated innate programs are differentially distributed across tumor-associated myeloid states

Given the heterogeneity and CAR-dependent remodeling of the myeloid compartment, we next asked whether a defined innate immune sensing axis could be resolved across tumor-associated myeloid states. We constructed a TLR7/8-associated transcriptional framework encompassing receptor expression, downstream signaling components, interferon-inducing transcription factors, antigen-presentation/costimulatory machinery, and broader myeloid response programs. Mapping these modules onto the myeloid UMAP revealed spatially restricted expression patterns, with discrete myeloid states exhibiting differential representation of sensing, signaling, interferon, and antigen-presentation components (**Supplementary Fig. 14A-C**).

**Figure 4.**
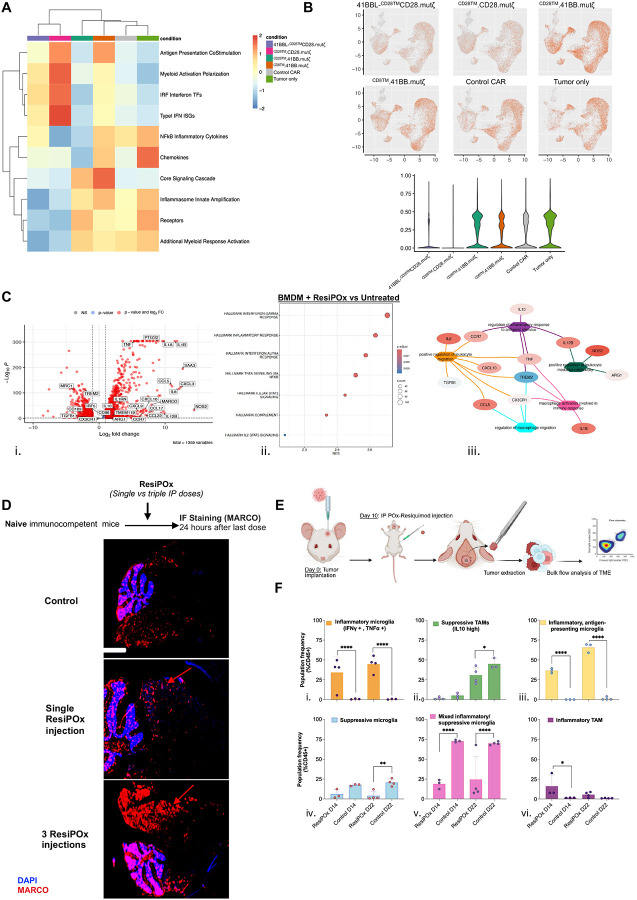
TLR7/8-associated signaling networks support targeting tumor-associated macrophages in the medulloblastoma microenvironment. A. Heatmap showing relative expression of the indicated TLR7/8-response associated genes across tumor-only and CAR-treated conditions. Columns represent treatment groups, and rows represent individual genes. Color intensity denotes scaled expression values, highlighting treatment-dependent differences in activation of the TLR7/8-associated inflammatory transcriptional program. B. Spatial distribution and abundance of TLR7/8-signature–positive myeloid cells across treatment conditions. Top, feature plots showing the UMAP distribution of cells expressing the TLR7/8 gene signature across tumor-only and CAR-treated groups, demonstrating treatment-associated differences in localization and enrichment of TLR7/8-programmed myeloid populations. Bottom, violin plot summarizing TLR7/8 signature scores across conditions. Each violin represents the distribution of per-cell module scores within the indicated treatment group, illustrating relative enrichment of the TLR7/8-associated transcriptional program following CAR T-cell therapy. C. Immunomodulatory effects of ResiPOx on BMDM demonstrated through endpoint-derived sequencing data i. Volcano plot showing differentially expressed genes associated with the myeloid transcriptional program of interest, highlighting selective enrichment of genes linked to inflammatory signaling, innate immune activation, and macrophage-associated responses. ii. Gene set enrichment summary plot showing pathway-level activation patterns associated with the same transcriptional comparison, including inflammatory signaling, interferon-related pathways, and innate immune response modules. Dot size reflects gene count within each pathway, and color denotes enrichment direction and magnitude. iii. Network visualization of selected differentially expressed genes and inferred functional interactions related to TLR7/8-associated innate immune signaling. Nodes represent individual genes, and edges indicate predicted or curated relationships among inflammatory mediators, macrophage-associated markers, and signaling intermediates, highlighting interconnected modules linked to cytokine signaling, antigen presentation, and immune-cell activation. D*. In vivo* assessment of macrophage responses following administration of ResiPOx in naïve immunocompetent mice. Left, experimental schematic showing single or repeated ResiPOx dosing followed by brain harvest and immunofluorescence analysis 24 hours after the final dose. Right, representative immunofluorescence images of brain sections stained for DAPI and MARCO, comparing untreated control mice with mice receiving one or three ResiPOx injections. E. Experimental schematic of the *in vivo* treatment workflow evaluating ResiPOx in the tumor setting. Mice were implanted intracranially with tumor cells, treated with ResiPOx according to the indicated schedule, and subsequently analyzed for tumor burden and TME composition. *Created in BioRender. Yaacoub, S. (2026)* https://BioRender.com/t7llgpy F. Quantification of intracellular (IC) and extracellular (EC/EEC) immune phenotypes from brain derived myeloid populations at day 4 (D14) and day 12 (D22) following ResiPOx treatment compared with untreated controls. IC analyses show increased inflammatory microglial and macrophage programs with concurrent loss of suppressive IL10/ARG1/AHR-associated states, while EC analyses reveal enrichment of inflammatory chemokine producing and antigen presenting microglial phenotypes and reduction of suppressive populations. Bars represent mean – SEM with individual biological replicates shown. (n=4 per condition; analysis was conducted using 2-way ANOVA with Sidak’s multiple comparisons test statistical significance is indicated as * p < 0.05, ** p < 0.01, *** p < 0.001, and **** p < 0.0001)

Notably, the most efficacious CAR designs (4–1BBL^CD28TM^CD28.mutζ and ^CD28TM^CD28.mutζ) were associated with relatively lower baseline TLR7/8 expression and signaling compared with other conditions, suggesting that productive antitumor immunity is linked to selective, state-dependent innate sensing rather than uniform activation of the TLR7/8 axis (Fig. 4A–B).

### Resiquimod acts as a targeted innate immunomodulator

To therapeutically engage the structured innate immune axis identified within the TME, we selected the TLR7/8 agonist resiquimod formulated in a poly(2-oxazoline) nanocarrier (ResiPOx) as an adjunct immunomodulatory strategy^35,37,41^ (**Supplementary Fig. 15A**). Because macrophages represent a major TLR7/8-expressing population within the TME, bone marrow–derived macrophages (BMDMs) were differentiated *in vitro* and polarized into M1- and M2-like states. Flow cytometric analysis confirmed expression of TLR7 and TLR8 across polarization conditions, supporting macrophages as direct targets of ResiPOx (**Supplementary Fig. 15B–C**).

Bulk RNA sequencing following ResiPOx exposure revealed a robust shift toward inflammatory macrophage activation, with upregulation of pro-inflammatory cytokines, chemokines, and costimulatory molecules and concomitant downregulation of markers associated with alternative activation and immunosuppression (Fig. 4C–i). Pathway-level analyses demonstrated coordinated induction of interferon- and inflammation-associated programs, including TNFα–NFκB, IFNα/γ, IL6–JAK–STAT3, and IL2–STAT5 signaling (Fig. 4C-ii–iii), consistent with targeted activation of innate immune signaling downstream of TLR engagement.

Importantly, ResiPOx did not directly affect CAR T-cell fitness or cytotoxicity. Murine CAR-expressing and non-transduced T-cells exhibited minimal TLR7 expression (**Supplementary Fig. 15D**), unchanged expansion kinetics, and preserved cytolytic activity across clinically relevant ResiPOx concentrations (**Supplementary Fig. 15E–F**), indicating that resiquimod acts primarily through myeloid modulation rather than direct CAR T-cell stimulation.

Finally, to assess CNS delivery and biological activity *in vivo*, na ve immunocompetent mice were treated with one or three doses of ResiPOx. Multidose administration resulted in increased brain penetration and enhanced MARCO staining compared with single-dose treatment, consistent with inflammatory macrophage polarization within the CNS (Fig. 4D).

### ResiPOx remodels the G3MB TME and Enhances CAR T-cell efficacy

In syngeneic G3MB models, ResiPOx treatment induced marked remodeling of the TME (Fig. 4E). High-dimensional spectral FACS revealed increased inflammatory microglia and antigen-presenting myeloid populations, accompanied by reductions in suppressive TAM and microglial subsets at both early and intermediate timepoints (Fig. 4F; **Supplementary Fig. 16A–B**). These innate changes were associated with enhanced activation and expansion of endogenous CD8^+^ T-cells, indicating coordinated innate–adaptive immune remodeling.

**Figure 5.**
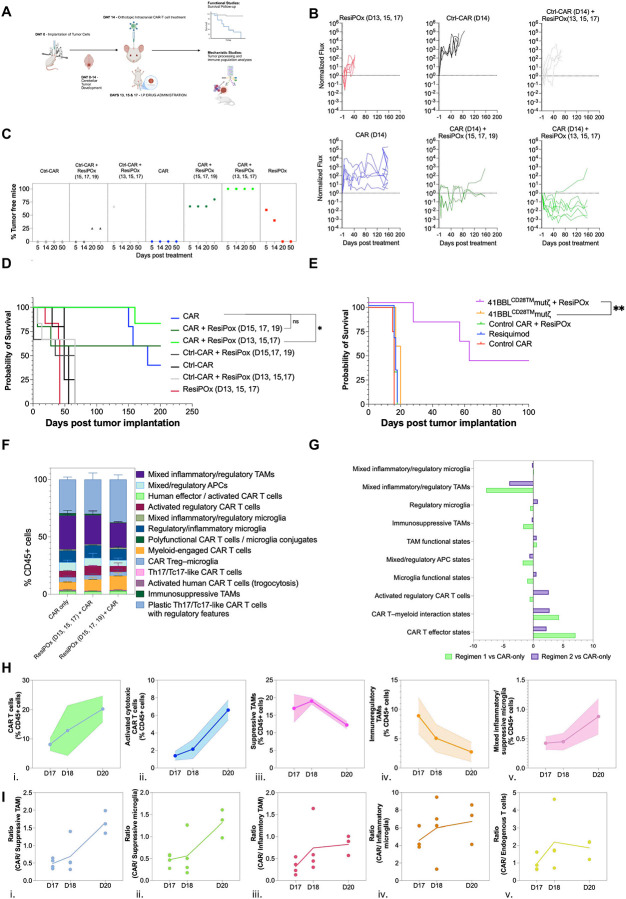
ResiPOx augments CAR T-cell antitumor activity *in vivo*. A. Schematic of the modified *in vivo* experimental workflow incorporating ResiPOx treatment into the orthotopic intracranial CAR T-cell model. Tumor cells were implanted intracranially on day 0 and allowed to establish in the cerebellum through day 14. CAR T-cells were administered intracranially on day 14, and ResiPOx was delivered by intraperitoneal injection on days 13, 15, and 17. Downstream studies included functional assessment by survival follow-up and mechanistic analyses of tumor and immune populations. B. Longitudinal bioluminescence imaging analysis of orthotopic D556-FFLuc tumor burden following treatment with the indicated CAR constructs, with or without ResiPOx. Plots show normalized flux over time for individual mice after treatment initiation. Comparative flux trajectories were used to assess treatment-associated changes in tumor burden, demonstrating reduced tumor progression in select combination-treated groups relative to corresponding single-treatment controls. C. Tumor-free survival following CAR T-cell therapy with or without ResiPOx. Dot plot showing the percentage of tumor-free mice over time after treatment across the indicated groups: control CAR, control CAR plus ResiPOx, CAR alone, CAR plus ResiPOx, and ResiPOx alone. Each symbol represents the proportion of tumor-free animals at the indicated time point, with group sizes shown in parentheses where applicable. Combination treatment with CAR and ResiPOx resulted in the highest proportion of tumor-free mice, supporting improved antitumor activity relative to monotherapy or control conditions. D. Kaplan–Meier survival analysis of NSG mice treated with control or B7-H3 CAR T-cells with or without ResiPOx. Survival curves comparing overall survival across the indicated treatment groups. Mice treated with B7-H3 CAR T-cells plus ResiPOx demonstrated the most durable survival benefit relative to control-treated cohorts, whereas CAR T-cell monotherapy showed intermediate activity. These data support enhanced antitumor efficacy with combined CAR T-cell therapy and innate immune modulation. (n=6 per treatment group; survival curves were determined using the log-rank (Mantel-Cox) test. * p<0.05; ** p<0.01; *** p<0.001; **** p<0.0001; ns, non-significant.) E. Kaplan–Meier survival analysis of syngeneic immunocompetent mice treated with the indicated conditions, including dual costimulatory domain CAR T-cells with or without ResiPOx. Combination treatment with 41BBL-^CD28TM^CD28.mutζ CAR T-cells plus Resiquimod significantly prolonged survival relative to CAR T-cell treatment alone, indicating that incorporation of the Resiquimod regimen substantially enhanced therapeutic efficacy *in vivo*. (n=5 per treatment group; survival curves were determined using the log-rank (Mantel-Cox) test. * p<0.05; ** p<0.01; *** p<0.001; **** p<0.0001; ns, non-significant.) F. Flow cytometric profiling of immune-cell composition and treatment-associated phenotypic shifts in endpoint NSG brain tumors. Stacked bar plot showing the relative abundance of immune cell populations identified using the extracellular and intracellular flow cytometry panels in brain tumors harvested at endpoint from NSG mice across the indicated treatment groups. Each bar represents the proportional composition of the analyzed CD45+ compartment, highlighting treatment-dependent differences in myeloid and lymphoid subset distribution. G. Waterfall plot summarizing the direction and magnitude of treatment-associated changes across the indicated immune populations and marker-defined subsets measured in the same endpoint samples. Together, these analyses define the endpoint immune landscape and demonstrate how treatment reshapes the intracranial tumor immune compartment in the NSG model when exposed to regimen 1 or 2 of ResiPOx. H. Early immune remodeling in immunocompetent intracranial tumors following ResiPOx and 4–1BBL-harboring CAR T-cell therapy. Longitudinal quantification of immune populations and marker-defined phenotypes from immunocompetent brain tumors harvested at early post-treatment time points days 17, 18, and 20 with ResiPOx in combination with 4–1BBL CAR T-cells. There are defined group-level trends over time for the indicated flow cytometric readouts, with shaded regions representing variability across samples. I. Early post-treatment dynamics of immune-cell populations in immunocompetent intracranial tumors following ResiPOx-based therapy. Trended scatter plots showing individual sample-level values for the indicated flow cytometric immune readouts from brain tumors harvested on days 17, 18, or 20 in the immunocompetent model. Each point represents a single tumor, plotted by collection time to visualize temporal evolution during the early post-treatment window, and lines indicate the fitted trend across time for each treatment condition. These analyses were performed in mice treated with ResiPOx combined with 4–1BBL CAR T-cells and reveal dynamic, feature-specific remodeling of the tumor immune compartment during the initial response phase. By displaying per-sample variability rather than group averages alone, these plots highlight the heterogeneity of early immune responses and provide higher-resolution evidence that innate immune modulation, with CAR T-cell therapy drives time-dependent changes in the intracranial TME.

We next evaluated whether ResiPOx could potentiate CAR T-cell therapy. In orthotopic xenograft G3MB models, combination treatment with ResiPOx and B7-H3 CAR T-cells resulted in superior tumor control compared with either modality alone (Fig. 5A–B). Critically, innate pre-conditioning with ResiPOx prior to CAR T-cell infusion (days 13, 15, and 17) achieved 100% tumor-free survival through day 50, whereas delayed ResiPOx administration following CAR T-cell delivery conferred only modest benefit (Fig. 5C–D).

This strategy was further validated using a human 4–1BBL-expressing B7-H3 CAR in combination with ResiPOx, which similarly produced pronounced antitumor activity (**Supplementary Fig. 17**). Importantly, in immunocompetent C57BL/6 mice, combination therapy also enhanced tumor control relative to 4–1BBL-expressing B7-H3 CAR T-cell treatment alone (Fig. 5E). Across CAR designs, the greatest therapeutic benefit was observed when ResiPOx was paired with 4–1BBL-expressing CARs, underscoring the importance of selective innate conditioning and optimized CAR architecture for maximizing CAR T-cell efficacy in G3MB.

### Time-dependent innate conditioning shapes CAR T-cell fate and CNS immune remodeling

We next investigated how the timing of innate immune conditioning influences CAR T-cell differentiation and immune remodeling within the CNS. In NSG mice, pre-conditioning with ResiPOx prior to CAR T-cell infusion preferentially enriched activated CAR T-cell effector states while reducing regulatory and suppressive CAR T-cell phenotypes at endpoint, coinciding with attenuation of suppressive myeloid transcriptional signatures (Fig. 5F–G). In contrast, delayed ResiPOx administration after CAR T-cell infusion triggered robust innate activation but failed to restore CAR T-cell effector programs, indicating that early myeloid conditioning is required to establish a permissive niche for durable CAR T-cell function.

Consistent with these findings, longitudinal immune profiling in immunocompetent G3MB-bearing mice revealed progressive CNS immune remodeling following combination therapy (**Supplementary Fig. 18A**). CAR T-cell frequencies increased between days 17 and 20, accompanied by expansion of activated CAR T-cell effector subsets (Fig. 5H). Endogenous Th1-polarized CD4^+^ and effector CD8^+^ T-cell populations also accumulated within the tumor. In parallel, combination therapy rapidly depleted intermediate suppressive TAMs and immunoregulatory microglial states, with sustained suppression over time, whereas ResiPOx monotherapy preserved higher frequencies of suppressive myeloid populations (**Supplementary Fig. 18B–D**).

To directly assess CAR T-cell infiltration relative to myeloid suppression, we quantified ratios of CAR T-cells to defined TAM subsets across post-treatment time points (Fig. 5I). The ratio of CAR T-cells to suppressive TAM and regulatory microglial populations increased progressively from day 17 to day 20, indicating that CAR T-cell accumulation outpaced persistence of immunosuppressive myeloid states (Fig. 5I-i–ii). In contrast, ratios relative to inflammatory myeloid subsets increased more modestly over the same interval (Fig. 5I-iii–iv), suggesting that ResiPOx promotes selective, time-dependent relief of immune suppression rather than indiscriminate myeloid expansion. Despite robust early expansion, CAR T-cell persistence remained a limiting factor, as reflected by declining CAR T-to-endogenous T-cell ratios over time (Fig. 5I-v).

Collectively, these data demonstrate that the timing of innate immune modulation is a critical determinant of CAR T-cell fate and CNS immune remodeling, with early, targeted myeloid conditioning driving durable effector programming and sustained antitumor immunity.

## Discussion:

This study addresses two fundamental barriers to effective CAR T-cell therapy in medulloblastoma: intrinsic limitations of CAR design and the profoundly suppressive, myeloid-dominant TME. Although multiple B7-H3 CAR constructs demonstrated potent antigen-dependent cytotoxicity *in vitro*, these differences failed to translate into durable survival benefit in immunocompetent intracranial models. These findings underscore the limitations of short-term killing assays as predictors of therapeutic efficacy in CNS tumors and reinforce the importance of immunocompetent systems that capture the dominant constraints on CAR performance, including persistence, trafficking, and suppression by endogenous immune populations^12^.

Among the CAR designs tested, the dual-costimulatory CD28/4–1BBL architecture conferred the greatest antitumor activity yet remained non-curative, indicating that CAR optimization alone is insufficient to overcome high-risk medulloblastoma in the absence of coordinated microenvironmental remodeling. Notably, transcriptomic and phenotypic profiling revealed that therapeutic efficacy was not associated with indiscriminate immune activation but instead correlated with selective reprogramming of tumor-associated myeloid states. Improved CAR performance aligned with relief of suppressive myeloid programs rather than amplification of inflammatory signaling per se, highlighting myeloid dominance as a central barrier to durable response^15^.

Across CAR constructs, baseline T-cell lineage distributions were largely preserved and did not predict efficacy. Instead, therapeutic benefit correlated with directional progression along a defined differentiation trajectory, moving from activation and proliferation toward terminal cytotoxic effector states. This trajectory was most clearly resolved in the 4–1BBL-expressing CAR, whereas intermediate-efficacy constructs accumulated in sustained cycling and metabolically stressed states without committing to effector differentiation. Low-performing CARs failed to initiate productive activation altogether. Together, these findings support a trajectory-based model of CAR T-cell efficacy, in which durable antitumor activity depends on successful resolution of activation and proliferative stress into terminal effector differentiation, rather than on the magnitude of activation alone. Importantly, these T-cell fate decisions were tightly coupled to CAR-dependent remodeling of the myeloid compartment, reinforcing the interdependence of lymphoid and innate immunity in the CNS TME.

These insights provided a mechanistic rationale for combining CAR T-cell therapy with ResiPOx, a nanoformulated TLR7/8 agonist designed to selectively engage innate immune circuits. ResiPOx robustly induced pro-inflammatory macrophage and microglial programs, enhanced antigen-presentation features, and suppressed immunoregulatory myeloid states within the medulloblastoma TME^37^. Crucially, ResiPOx did not augment CAR T-cell cytotoxicity in vitro, supporting a model in which its primary function is reprogramming of the neuroimmune niche rather than direct enhancement of T-cell killing. This distinction is central to understanding the observed synergy *in vivo*.

Accordingly, combination therapy significantly extended survival compared with CAR T-cells alone, and treatment timing emerged as a critical determinant of efficacy. Innate immune pre-conditioning prior to CAR infusion was markedly more effective than delayed modulation, indicating that early myeloid–T-cell interactions establish permissive or restrictive immune trajectories that shape subsequent CAR fate, persistence, and function. These data support a model in which durable CAR activity in medulloblastoma requires not only optimal CAR signaling architecture, but also early and coordinated remodeling of the neuroimmune microenvironment.

To our knowledge, this work represents one of the first demonstrations of nanoparticle-enabled TLR7/8-directed myeloid reprogramming combined with syngeneic CAR T-cell therapy in immunocompetent Group 3 medulloblastoma. This platform uniquely enables interrogation of both therapeutic efficacy and endogenous TME remodeling in a clinically relevant context^31,37,41^. Importantly, preclinical evaluation of ResiPOx in non-human primates has demonstrated favorable tolerability and induction of brain immune transcriptional programs that mirror those observed in murine tumor models^47^. Together with the absence of overt toxicity in our studies, these data inform key translational considerations, including dose feasibility, target-engaged innate activation in a physiologically relevant species, and early safety parameters necessary to advance this strategy toward clinical testing.

Several limitations warrant consideration. Available preclinical models incompletely recapitulate the full heterogeneity and developmental context of human medulloblastoma. The number of CAR architectures evaluated was necessarily finite, and additional designs may reveal distinct or superior immune trajectories. Finally, further work is required to delineate the precise cellular mediators of CAR–ResiPOx synergy and to rigorously define the safety boundaries of TLR7/8 pathway activation within the CNS. Nonetheless, these findings establish a conceptual framework in which effective CAR T-cell therapy for medulloblastoma requires both engineered T-cell optimization and early, targeted innate immune remodeling, providing a rational path forward for combinatorial immunotherapy in high-risk pBTs.

## Supplementary Material

Supplementary Files

This is a list of supplementary files associated with this preprint. Click to download.

- SupplementalMethodsSY.docx
- SupplementaryFigsSY.pdf

## Data Availability

The data generated and analyzed during this study are available from the corresponding author upon reasonable request. The reproducible code used to analyze the data and generate the figures for the manuscript can be found at https://github.com/childrens-bti/haydar-tme-cart-mb. Data is being deposited in GEO and will be assigned an accession number. Link for the manuscript data: https://doi.org/10.5281/zenodo.19955187 Link for the repo: https://github.com/childrens-bti/haydar-tme-cart-mb

## Key points:

- Dual CD28/4–1BB signaling improves CAR T efficacy in Group 3 medulloblastoma
- CAR architecture programs T cell and TAM states, linking the TME to response
- TLR7/8 agonism primes myeloid cells in the TME and potentiates CAR T-cell activity

## References

[1] Orr B. A. Pathology, diagnostics, and classification of medulloblastoma. Brain Pathol. 30, 664–678 (2020). 32239782 10.1111/bpa.12837 PMC7317787

[2] Adiamah M. MYC-dependent upregulation of the de novo serine and glycine synthesis pathway is a targetable metabolic vulnerability in group 3 medulloblastoma. Neuro-Oncol. 27, 237–253 (2025). 39377369 10.1093/neuonc/noae179 PMC11726242

[3] Mayr L., Azizi A. A., Gojo J. & Peyrl A. Medulloblastoma: Current Standard of Care and Future Treatment Opportunities. Pediatr. Drugs 28, 31–42 (2026). 10.1007/s40272-025-00726-1 PMC12864240 41120656

[4] Voskamp M. J. Immunotherapy in Medulloblastoma: Current State of Research, Challenges, and Future Perspectives. Cancers 13, (2021). 10.3390/cancers13215387 PMC8582409 34771550

[5] Shi X., Liu S., Sun X., Li Y. & Jiang M. Treatment Outcomes and Prognostic Factors for Patients With Medulloblastoma Having Defined Molecular Subtypes. Adv. Radiat. Oncol. 10, 101796 (2025). 40837387 10.1016/j.adro.2025.101796 PMC12361598

[6] Akhavan D. CAR T cells for brain tumors: Lessons learned and road ahead. Immunol. Rev. 290, 60–84 (2019). 31355493 10.1111/imr.12773 PMC6771592

[7] Fonkoua L. A. K., Sirpilla O., Sakemura R., Siegler E. L. & Kenderian S. S. CAR T cell therapy and the tumor microenvironment: Current challenges and opportunities. Mol. Ther. - Oncolytics 25, 69–77 (2022). 35434273 10.1016/j.omto.2022.03.009 PMC8980704

[8] Landry A. P., Dunn G. P. & Lim M. Current landscape of immunotherapy for CNS tumors. Neuro-Oncol. Adv. 7, iv1–iv3 (2025). 10.1093/noajnl/vdaf118 PMC12418591 40933035

[9] Zhang X., Zhu L., Zhang H., Chen S. & Xiao Y. CAR-T Cell Therapy in Hematological Malignancies: Current Opportunities and Challenges. Front. Immunol. Volume 13–2022, (2022). 10.3389/fimmu.2022.927153 PMC9226391 35757715

[10] Lin Y.-J., Mashouf L. A. & Lim M. CAR T Cell Therapy in Primary Brain Tumors: Current Investigations and the Future. Front. Immunol. Volume 13–2022, (2022). 10.3389/fimmu.2022.817296 PMC8899093 35265074

[11] Guzman G., Pellot K., Reed M. R. & Rodriguez A. CAR T-cells to treat brain tumors. Brain Res. Bull. 196, 76–98 (2023). 36841424 10.1016/j.brainresbull.2023.02.014

[12] Ferreras C. Facing CAR T Cell Challenges on the Deadliest Paediatric Brain Tumours. Cells 10, (2021). 10.3390/cells10112940 PMC8616287 34831165

[13] Haydar D. Cell-surface antigen profiling of pediatric brain tumors: B7-H3 is consistently expressed and can be targeted via local or systemic CAR T-cell delivery. Neuro-Oncol. 23, 999–1011 (2020). 10.1093/neuonc/noaa278 PMC8168826 33320196

[14] Majzner R. G. GD2-CAR T cell therapy for H3K27M-mutated diffuse midline gliomas. Nature 603, 934–941 (2022). 35130560 10.1038/s41586-022-04489-4 PMC8967714

[15] Kalathoor S. Myeloid cell heterogeneity in the tumor microenvironment and therapeutic implications for childhood central nervous system (CNS) tumors. J. Neuroimmunol. 374, 578009 (2023). 36508930 10.1016/j.jneuroim.2022.578009

[16] Meza Pacheco M. F. & Tai L.-H. Reprogramming the tumor microenvironment to boost adoptive T cell therapy. Front. Immunol. Volume 16–2025, (2025). 10.3389/fimmu.2025.1677548 PMC12589096 41208982

[17] Kennedy B. C. Tumor-Associated Macrophages in Glioma: Friend or Foe? J. Oncol. 2013, 486912 (2013). 23737783 10.1155/2013/486912 PMC3664503

[18] Akhavan D. CAR T cells for brain tumors: Lessons learned and road ahead. Immunol. Rev. 290, 60–84 (2019). 31355493 10.1111/imr.12773 PMC6771592

[19] Guzman G., Pellot K., Reed M. R. & Rodriguez A. CAR T-cells to treat brain tumors. Brain Res. Bull. 196, 76–98 (2023). 36841424 10.1016/j.brainresbull.2023.02.014

[20] Binder Z. A., Bagley S. J., Foster J. B. & O’Rourke D. M. The development of CAR T cells for patients with CNS malignancies. Nat. Rev. Clin. Oncol. 23, 137–150 (2026). 41326789 10.1038/s41571-025-01102-1 PMC12854404

[21] Haydar D. CAR T-cell Design-dependent Remodeling of the Brain Tumor Immune Microenvironment Modulates Tumor-associated Macrophages and Anti-glioma Activity. Cancer Res. Commun. 3, 2430–2446 (2023). 37971169 10.1158/2767-9764.CRC-23-0424 PMC10689147

[22] Lee M. J., Cichocki F. & Miller J. S. Chimeric antigen receptor therapies: Development, design, and implementation. J. Allergy Clin. Immunol. 156, 70–80 (2025). 40220909 10.1016/j.jaci.2025.04.005 PMC12229777

[23] Keller C. W., Mundt S. & Segal B. M. Editorial: CNS myeloid cell function in health and disease. Front. Immunol. Volume 15–2024, (2024). 10.3389/fimmu.2024.1459138 PMC11288962 39086488

[24] Herz J., Filiano A. J., Wiltbank A. T., Yogev N. & Kipnis J. Myeloid Cells in the Central Nervous System. Immunity 46, 943–956 (2017). 28636961 10.1016/j.immuni.2017.06.007 PMC5657250

[25] Frosch M. & Prinz M. Niche-specific therapeutic targeting of myeloid cells in the central nervous system. Immunity 58, 1101–1119 (2025). 40324377 10.1016/j.immuni.2025.03.016

[26] Amann L., Masuda T. & Prinz M. Mechanisms of myeloid cell entry to the healthy and diseased central nervous system. Nat. Immunol. 24, 393–407 (2023). 36759712 10.1038/s41590-022-01415-8

[27] Herz J., Filiano A. J., Wiltbank A. T., Yogev N. & Kipnis J. Myeloid Cells in the Central Nervous System. Immunity 46, 943–956 (2017). 28636961 10.1016/j.immuni.2017.06.007 PMC5657250

[28] Crotty E. E. Medulloblastoma recurrence and metastatic spread are independent of colony-stimulating factor 1 receptor signaling and macrophage survival. J. Neurooncol. 153, 225–237 (2021). 33963961 10.1007/s11060-021-03767-x PMC8248272

[29] Mayr L., Azizi A. A., Gojo J. & Peyrl A. Medulloblastoma: Current Standard of Care and Future Treatment Opportunities. Pediatr. Drugs 28, 31–42 (2026). 10.1007/s40272-025-00726-1 PMC12864240 41120656

[30] Hu A. Harnessing innate immune pathways for therapeutic advancement in cancer. Signal Transduct. Target. Ther. 9, 68 (2024). 38523155 10.1038/s41392-024-01765-9 PMC10961329

[31] Frega G. Trial Watch: experimental TLR7/TLR8 agonists for oncological indications. OncoImmunology 9, 1796002 (2020). 32934889 10.1080/2162402X.2020.1796002 PMC7466852

[32] Fitzgerald K. A. & Kagan J. C. Toll-like Receptors and the Control of Immunity. Cell 180, 1044–1066 (2020). 32164908 10.1016/j.cell.2020.02.041 PMC9358771

[33] Alvarez-Arellano L. High expression of Toll-like receptor 7 is a survival factor in pediatric medulloblastoma. Childs Nerv. Syst. 37, 3743–3752 (2021). 34480601 10.1007/s00381-021-05347-w

[34] Mukherjee S. Toll-like receptor-guided therapeutic intervention of human cancers: molecular and immunological perspectives. Front. Immunol. Volume 14–2023, (2023). 10.3389/fimmu.2023.1244345 PMC10562563 37822929

[35] Vinod N. High-capacity poly(2-oxazoline) formulation of TLR 7/8 agonist extends survival in a chemo-insensitive, metastatic model of lung adenocarcinoma. Sci. Adv. 6, eaba5542 (2020). 10.1126/sciadv.aba5542 PMC7299629 32596460

[36] Zhang X. Cell microparticles loaded with tumor antigen and resiquimod reprogram tumor-associated macrophages and promote stem-like CD8+ T cells to boost anti-PD-1 therapy. Nat. Commun. 14, 5653 (2023). 37704614 10.1038/s41467-023-41438-9 PMC10499806

[37] Rodell C. B. TLR7/8-agonist-loaded nanoparticles promote the polarization of tumour-associated macrophages to enhance cancer immunotherapy. Nat. Biomed. Eng. 2, 578–588 (2018). 31015631 10.1038/s41551-018-0236-8 PMC6192054

[38] Vo B. T. Inactivation of Ezh2 Upregulates Gfi1 and Drives Aggressive Myc-Driven Group 3 Medulloblastoma. Cell Rep. 18, 2907–2917 (2017). 28329683 10.1016/j.celrep.2017.02.073 PMC5415387

[39] Northcott P. A. Enhancer hijacking activates GFI1 family oncogenes in medulloblastoma. Nature 511, 428–434 (2014). 25043047 10.1038/nature13379 PMC4201514

[40] Jin S. Inference and analysis of cell-cell communication using CellChat. Nat. Commun. 12, 1088 (2021). 33597522 10.1038/s41467-021-21246-9 PMC7889871

[41] Lu R. Formulation and preclinical evaluation of a toll-like receptor 7/8 agonist as an anti-tumoral immunomodulator. J. Controlled Release 306, 165–176 (2019). 10.1016/j.jconrel.2019.06.003 PMC6679596 31173789

